# Natural Radioactivity Assessment and Radiation Hazards of Pegmatite as a Building Material, Hafafit Area, Southeastern Desert, Egypt

**DOI:** 10.3390/toxics10100596

**Published:** 2022-10-09

**Authors:** El-Afandy H. Adel, Samia H. Taha, Osama A. Ebyan, Wafaa M. Rashed, Mohamed G. El-Feky, Mohammed S. Alqahtani, Korany A. Korany, Mohamed Y. Hanfi

**Affiliations:** 1 Contracts Sector, Nuclear Materials Authority, Cairo P.O. Box 530, Egypt; 2 Department of Physics, Faculty of Science, Al Azhar University, Cairo P.O. Box 11884, Egypt; 3 Department of Physics, Faculty of Science, New Vally University, Alkharja 72511, Egypt; 4 Department of Radiological Sciences, College of Applied Medical Sciences, King Khalid University, Abha 61421, Saudi Arabia; 5 BioImaging Unit, Space Research Centre, Department of Physics and Astronomy, University of Leicester, Leicester LE1 7RH, UK; 6 Department of Basic Sciences, Common First Year Deanship, Jouf University, Sakaka P.O. Box 214, Saudi Arabia; 7 Institute of Physics and Technology, Ural Federal University, St. Mira 19, Yekaterinburg 620002, Russia

**Keywords:** natural radioactivity, south-eastern desert, pegmatite, alpha index, radiation hazards

## Abstract

Sixty-seven sites of Hafafit pegmatite from the Southeastern Desert of Egypt were investigated radiometrically in the field using an in situ γ-ray spectrometer to determine eU, eTh, and K contents. The obtained results ranged from 0.4 to 6 ppm for eU with a mean value of 2.5 ppm, from 0.2 to 32 ppm for eTh with a mean value of 6.7 ppm, and from 0.7% to 5.4% for K with a mean value of 3.3%. Consequently, the radiological effects from these rocks were estimates by determination of the environmental parameters: gamma activity concentration index Iγ, external hazard index H_ex_, internal hazard index H_in_, external absorbed dose rates in outdoor, and external absorbed dose rates in indoor air. The results obtained in this study showed that values U, Th, and K lie in the range of the acceptable world values. In addition, the calculated radiation hazard parameters (Iγ, H_ex_, and H_in_) have values lower than the world values, while the calculated external absorbed dose rates (D_air_) have values higher than the world and Egyptian permissible levels.

## 1. Introduction

Naturally occurring radioactive materials (NORM) can be found in a variety of places in the environment, including rocks, soil, water, and air. Because natural radionuclides are a result of the Earth’s origin, there are no solutions to eliminate their presence. To estimate the impacts of radiation exposure from both terrestrial and extraterrestrial sources, knowledge of radionuclide distribution and radiation levels in the environment is required. Despite the fact that these radionuclides are widely distributed, their concentrations are influenced by local geological conditions, which differ from one location to the next [1,2].

These radionuclides expose people to radiation both outside and inside their homes. Gamma radiation from the ^238^U and ^232^Th series isotopes, as well as 40K causes external exposure, but inhalation of ^222^Rn, ^220^Rn, and their short-lived progeny, which produce alpha particles, causes internal exposure [3]. Radionuclides activity concentrations measurement in building materials is essential in assessing population exposure, as most individuals spend 80% of their time indoors [4].

Pegmatite is a plutonic igneous rock with unusually coarse grains [5]. Granites have nearly the same mineralogical composition of pegmatite. K-feldspar (either orthoclase or microcline), quartz, and a few other minerals make up the majority of pegmatite. Tourmaline, lepidolite, topaz, cassiterite, fluorite, beryl, and other metals are frequent in complex pegmatite, and they have commercial significance.

The majority of NORM are found in rocks and soils in amounts that are safe for humans and the environment [5]. However, due to geological evolution, some regions have relatively high natural concentrations of U, Ra, and Th. In locations with high background levels, both natural and artificial rapid radionuclide mobilization occurs [6]. U and Th are long-lived radioactive isotopes that produce a variety of radioactive progeny products that can be hazardous to public health and the environment. Natural radiation accounts for the majority of overall radiation exposure in the general population. To assess the possible environmental hazard, designate the limit of areas with high natural background, and calculate the cleanup level, as radioactive background levels must be quantified [5,6,7,8].

As pegmatite rocks can be used as interior (decorative aggregates, flooring, and interior decoration) or exterior (building stone, facing stone, and paving stone) uses in industries and building construction, the use of building materials with above-average levels of natural radioactivity can significantly increase population radiation exposure. As a result, it is crucial to look into the radiation concerns provided by naturally occurring radionuclides in pegmatite.

The present study aimed to investigate the radioactivity and mineralogy of granites with the determination of their environmental impacts. The current study maps radioactive background levels in the surrounding environment and proposes a radiological assessment program in the Hafafit region in the southeastern desert of Egypt. This map will be used to examine any changes in radioactivity background levels as a result of geological processes or other radiation-related factors. The present work aimed to assess the exposure to gamma rays from the granitic rocks. The determination of radioactive danger is made by the estimation of some of the radiological hazard’s parameters.

## 2. Materials and Methods

**Figure 1:**
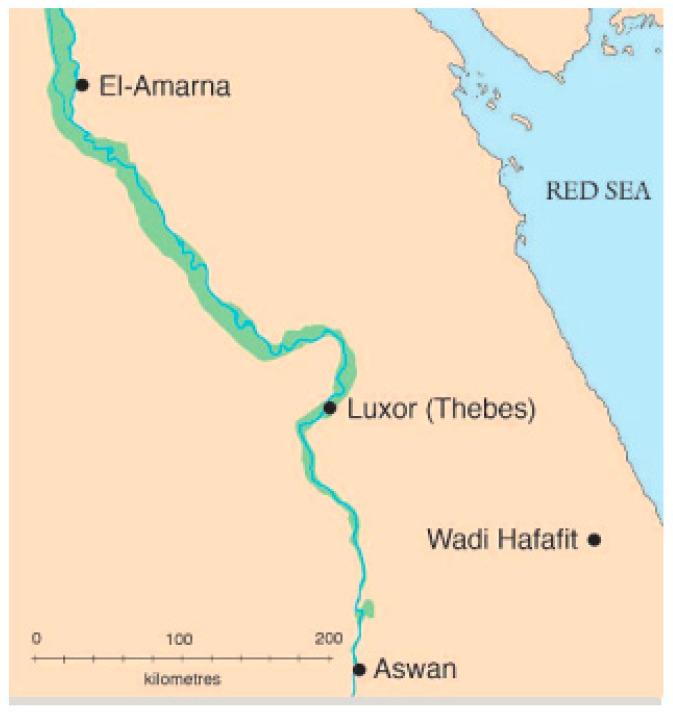
Location map of Hafafit area.

A total of 67 Hafafit pegmatite sites in the Southeastern Desert of Egypt (Figure 1) were investigated for natural radioactivity owing to eU (ppm), eTh (ppm), and K (%).

The quantities of radioactive elements (^238^U, ^232^Th, and ^40^K) in the pegmatite samples were monitored using a scintillating NaI (Tl) gamma-ray analyzer with a crystal phase of 7.6 cm × 7.6 cm. A low background measurement environment was ensured by placing the detector at an arrangement that was enclosed in a cylindrical lead shield with a diameter of 15.7 cm, a length of 20.5 cm, and a thickness of 3.7 cm, with an attenuation factor of 0.16 (stopping around 84% of input photons) for 2.6 MeV gamma-rays. A spectroscopic amplifier and a multi-channel analyzer were part of the pulse processing and data analysis system, which was linked to an IBM-compatible computer. The software computer that was attached to the pulse information and better estimation technique had a multi-channel analyzer and a spectroscopic amplifier. The γ–energies of the detection are, [^214^Bi: 1.764 MeV (I γ = 15.30%)] for ^226^Ra, [^208^Tl: 2.614 MeV (I γ = 99.754%)] for ^232^Th, and 1.460 MeV (I γ = 10.66%), for ^40^K, respectively [9,10]. Certified standard materials, like RGU-1 for ^238^U, RGTh-1 for ^232^Th, and RGK-1 for ^40^K, are utilized, and their pulverized quantities are comparable to individuals of the construction materials [11]. The container design was designed according to the assumption that the radioactive isotopes in the monitoring samples in the monitoring samples are uniformly distributed. Before the detection, the background was detected using an empty container, which was measured in the same manner and geometry of the samples. The background spectra were employed to prepare the area of the γ-spectrum of detected isotopes. The minimum detectable activity (MDAs) of 2, 4, and 12 Bq kg^−1^ are used for ^238^U, ^232^Th, and ^40^K, respectively, in samples recorded up to 2000 s. The overall uncertainty of the levels of radiation was calculated using the deviation equation for regular and stochastic misspecification. Systematic inaccuracies of 0.5 to 2 percent and randomness of up to 5% can be found in the radioactivity readings during the efficiency calibration [12]. After the detection of activity concentrations of ^238^U, ^232^Th, and ^40^K, the radiological variables are estimated according to Table 1.

## 3. Results

### 3.1. Radioactivity Measurement

**Figure 2:**
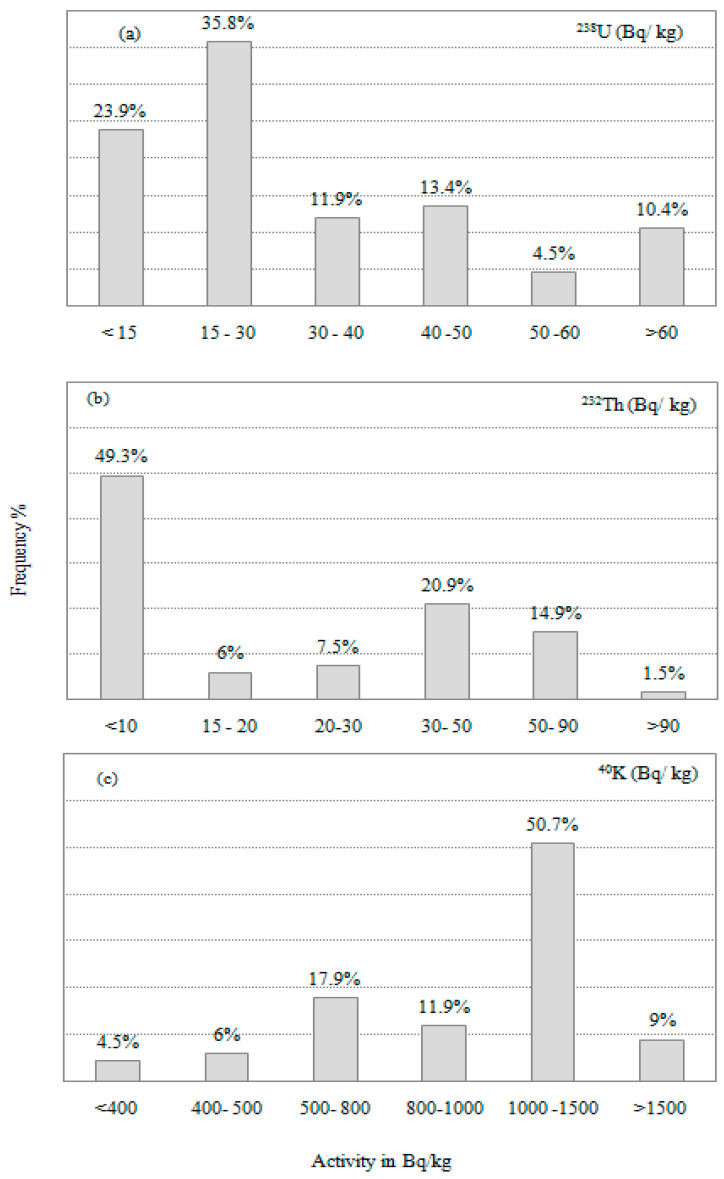
Frequency distributions of activity concentrations (Bq/kg) for pegmatite from Hafafit (**a**) ^238^U (**b**) ^232^Th and (**c**) ^40^K.

Table S1 showed the distribution of radionuclides identified in rock samples, including ^238^U (ppm), ^232^Th (ppm), and ^40^K (percent). The radioelement concentration in pegmatite ranged from 0.4 to 6 ppm for eU with a mean value of 2.5 ppm, from 0.2 to 32 ppm for eTh with a mean value of 6.7 ppm, and from 0.7 percent to 5.4% for K with a mean value of 3.3%. These results are lower than those reported for the Earth’s crust, which are 2.9 ppm, 10.8 ppm, and 2.7%, respectively [14], and the recommended values for safety used as building materials, which are 4.1 ppm, 12.3 ppm, and 1.6% for eU, eTh, and K, respectively [15], except in the case of K, which has a value higher. The frequency distribution in the examined samples suggested that low radionuclides contents except in the case of K (Figure 2).

**Figure 3:**
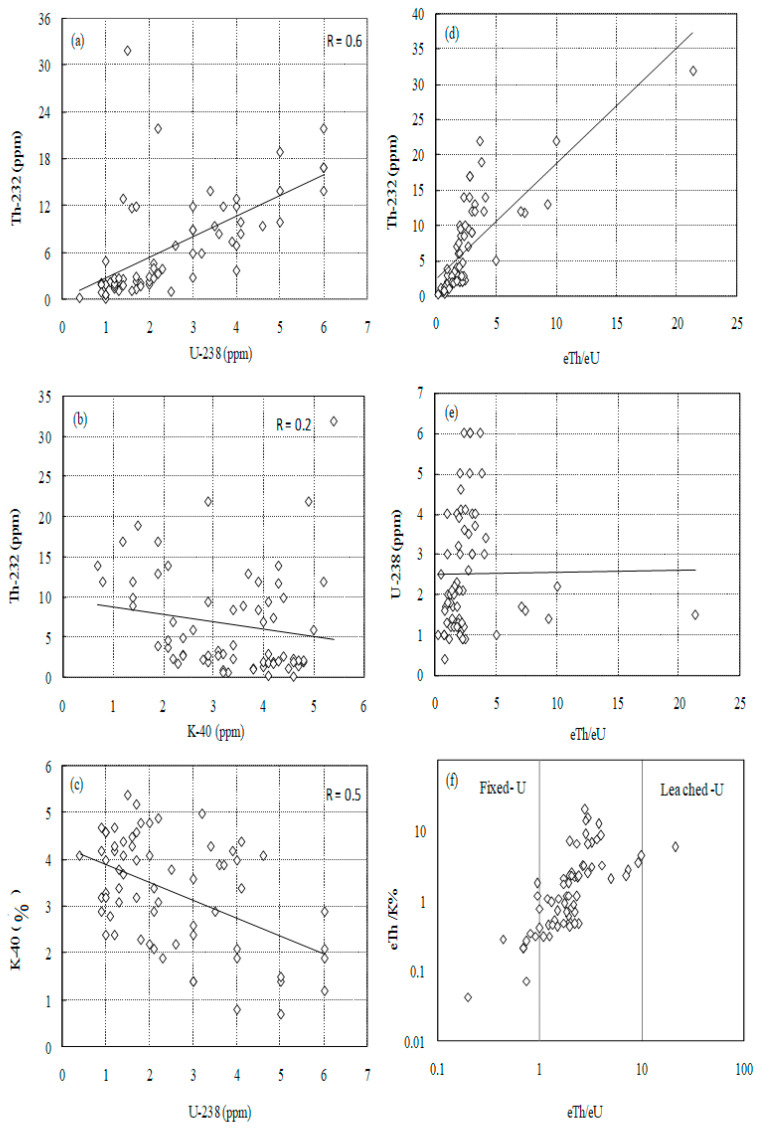
The radioactive correlations, (**a**) eTh vs. eU, (**b**) eTh vs. K% and (**c**) K vs. eU (**d**) eTh vs. eTh/eU, (**e**) eU vs. eTh/eU and (**f**) eTh/K% vs. eTh/eU for Hafafit pegmatite.

In Figure 3, the relationships between eU, eTh, and K were diagrammed and illustrated. We may deduce from the figure that Th and U have a positive association (R = 0.6) (Figure 3a), whereas K and whomsoever Th and U have an inverse relationship (R = 0.2 and 0.5, respectively) as shown in Figure 3b,c.

The amount of U remobilization that has happened within the magmatic plutons is indicated by variation diagrams of eU and eTh with their ratios [16,17]. The ratio of eTh to eTh/eU shows a rising trend. In the case of the connection between eU and the eTh/eU ratio (Figure 3d,e), respectively, an undefined relationship was seen. The relationship between Th/K and Th/U suggests that pegmatite samples are in the leached-U sector (Figure 3f).

As revealed in Table 2 the mean data of ^238^U, ^232^Th, and ^40^K activity concentrations are 30.8 ± 18.4, 27.3 ± 26 and 1045.5 ± 366.9 Bq kg^−1^, respectively; ^238^U, and ^232^Th are lower than the recommended worldwide average 33, 45 Bq kg^−1^ [18], while ^40^K activity concentrations are higher than worldwide average 412 Bq/kg [17,18]. The values of ^238^U activity concentrations altered between 4.9 and 74.1 Bq kg^−1^. The Min and Max values of ^232^Th are 0.8 and 129.9 Bq kg^−1^, respectively. Moreover, the variation of ^40^K values altered from 219.1 to 1690.2 Bq kg^−1^. The highest values of activity concentrations of ^238^U, ^232^Th, and ^40^K were recorded in the investigated pegmatitepegmatites due to the high radioactivity of altered pegmatite is referred to the occurrence of zircon, monazite, allanite, sphene and apatite, furthermore thorite, fergusonite, samarskite, columbite, xenotime, apatite, and fluorite. The descriptive statistics are performed to show the distribution of values, asymmetry nature of distribution and its Peakness for ^238^U, ^232^Th, and ^40^K, as well as the variability (Table 2).

**Figure 4:**
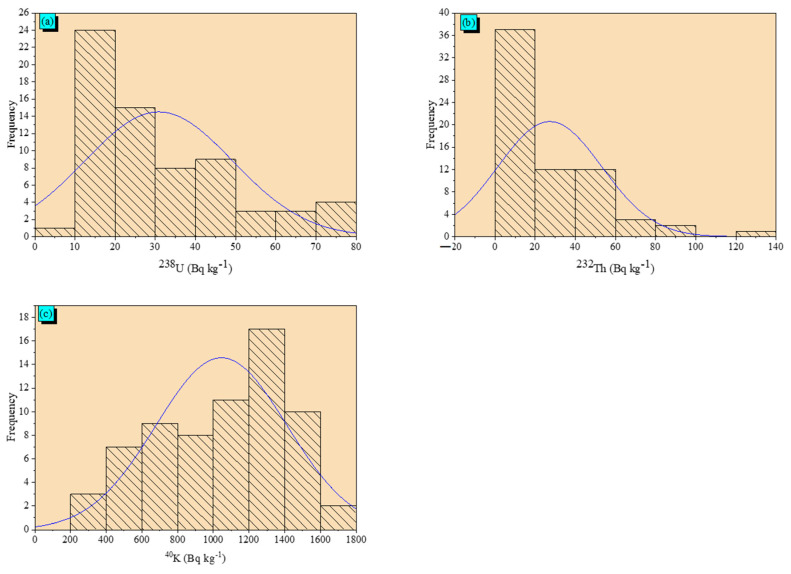
Frequency distribution of (**a**) ^238^U, (**b**) ^232^Th, and (**c**) ^40^K activity concentrations in studied area.

Among the statistical parameters are the skewness, kurtosis, and coefficient of variance. Positive skewness numbers represent the asymmetrical distribution’s head, while negative numbers represent its tail. As a result, asymmetry can be seen in the distributions of ^238^U and ^232^Th activity concentrations, while the asymmetric distribution of ^40^K activity concentrations is observed with the tail. The kurtosis values show where the probability distribution peaked. The kurtosis value for the ^232^Th activity concentrations in the area under study is +ve, and the likelihood of distribution is at its apex. The flatness of probability distribution of ^238^U and ^40^K activity concentrations is observed (–0.16 and –0.77, respectively). As clarified in Table 2, the coefficient of variance (CV) was offered in Table 2 with the high values 60% and 95% for ^238^U and ^232^Th, respectively, while the moderate variability is identified for ^40^K (35%) in the examined area. The variation is linked to the pegmatites in the investigated areas including the ^238^U and ^232^Th host minerals. Figure 4a–c displays the ^238^U, ^232^Th, and ^40^K activity concentrations distributions in the investigated area. The normal distribution is predicted for ^40^K activity concentrations in the studied area, while the multimodality distribution of ^238^U and ^232^Th is predicted.

The mean values of ^238^U, ^232^Th and ^40^K in granitic rocks samples are compared to the previous investigations (Table 3). The comparison shows that the geological characterization of the analyzed sites affects the activity concentrations of ^238^U, ^232^Th and ^40^K.

Table 4 illustrates that Ra_eq_ values for pegmatite samples alternate between 88 and 334 Bq kg^−1^ with a mean value of 150 Bq kg^−1^.The mean value of H_in_ and H_ex_ are 0.55 and 0.4, which are lower than the exceeded level. The values range (H_in_ and H_ex_) are changed from 0.3 to 1 and 0.2 to 0.9, respectively. The H_in_ and H_ex_ mean values in all pegmatites samples display there is no significant negative effects, i.e., these values are found to be lower than the reference level of the unit [28]. Furthermore, the highest H_in_ and H_ex_ values of pegmatite samples in the considered area may be contributed significant health impacts accompanying with gamma-rays, radon gas and its decay products. Moreover, the pegmatites that have been investigated the most indicate that they cannot be used as building and interior ornamental elements in dwellings.

The (D_air_) values of the studied pegmatites samples altered from 44.2 to 156.3 nGy h^−1^ with the mean value of 74 nGy h^−1^. The mean value of (D_air_) in the area under study exceeded the mean worldwide value—59 nGy/h [18]. This demonstrates that the pegmatites in the research region are unsuitable for use as construction materials or in other types of infrastructure.

Table 4 depicts the (AED_out_) values ranging between 0.05 and 0.19 mSv y^−1^ with the mean value of 0.09 mSv y^−1^, which is comparable with the approved worldwide value 0.07 mSv y^−1^ [18]. Moreover, the mean (AED_in_) value is 0.36 mSv y^−1^, which is lower than worldwide value of 0.41 mSv y^−1^ [18]. The rates of are AED_in_ values are in between 0.22 and 0.77 mSv y^−1^. Heavy minerals found in pegmatites, such as monazite, uraninite, and thorianite, can be blamed for the high doses. Deoxyribonucleic acid (DNA) in genes, cancer, and the degeneration of tissues are only a few of the negative health effects that long-term exposure to large amounts of this substance can have [29]. The annual gonadal dose equivalent (AGDE) varies in between 0.33 and 1.13 mSv with average value 0.54 mSv, much greater than the permissible value 0.33 mSv [17]. The adverse health effects will be induced due to exposure a long time to emitted gamma of the studied pegmatites through their life years. The exposure can be achieved as the application of the pegmatites various building materials and infrastructures fields. This can be predicated owing to the (ELCR) values alternating from 0.13 × 10^−3^ to 2.86 × 10^−3^ with a mean value of 0.49 × 10^−3^, which is suppressed the permissible value (0.29 × 10^−3^) [30].

### 3.2. Statistical Approach

#### 3.2.1. Pearson Correlation Analysis (PC)

The present study uses Pearson correlation to find strong connections and linear relationships among activity concentrations of radionuclides and radiological hazard indicators in pegmatite samples. According to the PC, the linear relationship between the analyzed parameters was categorized into four groups; the first is weak (0.00–0.19) correlation, the second is moderate (0.2–0.39) correlation, the third is strong (0.4–0.79) correlation, and the fourth is very strong (0.8–1.00) correlation [31]. Positive correlations are found among all of the observed parameters, as shown in Table 5. It shows that the radionuclides in the samples under investigation come from natural sources and that their geographic variation in the environment is undisturbed by other factors. A moderate correlation between ^238^U and ^232^Th activity concentrations in the studied pegmatites samples is enrolled. This indicates the existence of ^238^U and ^232^Th in the examined pegmatites from the natural chains. At the same time, a weak correlation is predicted between ^40^K and both ^238^U and ^232^Th. The correlations are very strong regarding the relations between ^232^Th and the variables of radiological hazards. The ^232^Th are mainly contributed to the radiological dangers and the risk linked to the emitted gamma rays from radioactive series in the pegmatite samples.

#### 3.2.2. Hierarchical Cluster Analysis (HCA)

**Figure 5:**
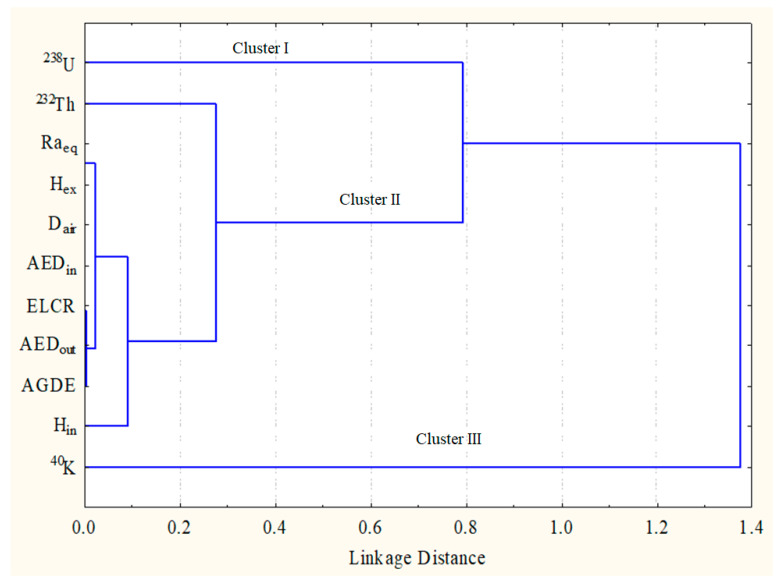
The clustering analysis of the radiological parameters in studied area.

Ward’s approach was used to achieve cluster analysis in this study. Ward’s approach links radioactive activity concentrations and radiological variables that estimate the Euclidean distance between them [31,32]. Figure 5 reveals two main clusters that are plotted in the dendrogram of the examined data. Cluster I includes ^238^U, which correlated with cluster II, which is composed of ^232^Th, Ra_eq_, H_ex_, H_in_, D_air_, AEDout, AED_in_, AGDE and ELCR. While Cluster III includes ^40^K and the remaining radiological characteristics according to HCA, the pegmatite’s radioactivity is connected to radioactive concentrations, particularly those of uranium and thorium. It is shown that the HCA data and the Pearson correlation agree.

#### 3.2.3. Principal Component Analysis (PCA)

**Figure 6:**
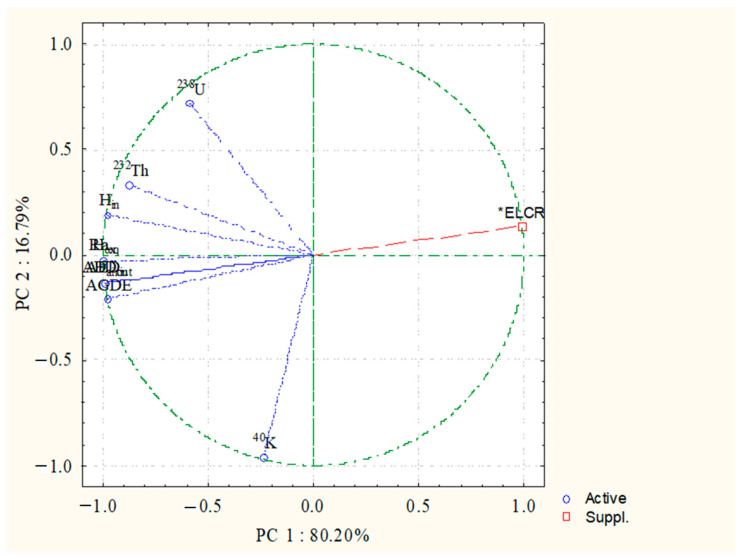
Principal component analysis (PC1 and PC2) for radiological data at studied area.

In this study, the matrix correlation between a number of components was determined using the PCA and varimax rotations. Figure 6 illustrates the components PC1 and PC2. In the PC1 loading linked with all radiological parameters, the activity concentrations of ^238^U and ^232^Th have a high loading. Overall, 80.20% of the variation is explained. Therefore, ^238^U and ^232^Th were the main sources of naturally occurring radioactivity in the pegmatite at the study area. In the PC2 load, however, ^40^K shows weak negative loading. The variance explained is 16.79%. As can be seen, the loading variance is negative, indicating that potassium has no influence on the radiation exposure grade. According to the PC analysis, the radioactive database’s overall explained variance was 96.99%, therefore the results were promising [28,33].

## 4. Conclusions

The novelty of this work is to assess the level of radioactivity in pegmatite rocks applied as building materials, such as ornamental stones, and in various infrastructure fields. The mean activity concentrations are 30.8 ± 18.4, 27.3 ± 26 and 1045.5 ± 366.9 Bq kg^−1^ for ^238^U, ^232^Th and ^40^K, respectively. Furthermore, all radiological hazard parameters for the investigated pegmatite samples were evaluated, and lower and comparable values with the acceptable levels were found. This is referred to the alteration of radioactive bearing minerals like zircon, allanite, monazite, sphene and apatite, etc., in the investigated pegmatite rocks. The statistical study was carried out to show that the radiological hazard parameters are linked to the thorium and its minerals. Pegmatite rocks include the radioactive minerals. As a result, the pegmatite rocks in the study area may be hazardous to human health and are unsuitable for use in various infrastructures, particularly as construction.

## Figures and Tables

**Table 1:** Important radiological parameters and indices [13].

Parameter	Definition	Formula
Ra_eq_	The radium equivalent content (Ra_eq_) is the radioactive parameter applied widely in radiation health hazards. The data of Ra_eq_ must be less than 370 Bq kg^−1^, which keeps the AED for the public lower than one mSv. The Ra_eq_ can be detected by the following formula.	Ra_eq_ (Bq kg^−1^) = A_Ra_ + 1.43 A_Th_ + 0.077 A_K_
D (nGy/h)	The radioactive factor known as the absorbed dose rate was used to evaluate the effect of gamma radiation at a distance of 1 m from radiation sources in the air owing to the concentrations of ^238^U, ^232^Th, and ^40^K.	D_air_ (nGy h^−1^) = 0.430 A_U_ + 0.666 A_Th_ + 0.042 A_K_
AED_out_	An element of radioactivity called the yearly effective dose is used to gauge radiation exposure levels over a fixed period of time (1 year).	AED_out_ (mSv/y) = D_air_ (nGy/h) × 0.2 * 8760 (h/y) × 0.7 (Sv/Gy) × 10^−6^ (mSv/nGy)
AED_in_		AED_in_ (mSv/y) = D_air_ (nGy/h) × 0.8 × 8760 (h/y) × 0.7 (Sv/Gy) × 10^−6^ (mSv/nGy)
H_ex_	The radiological parameters used to evaluate the risk of gamma radiation are known as the external hazard index. When radon and its decay products are exposed internally, the internal hazard index is used.	$H_{\mathrm{ex}}=\frac{A_{U}}{370}$ $+\frac{A_{Th}}{259}$ $+\frac{A_{K}}{4810}$
H_in_		$H_{\mathrm{in}}=\frac{A_{U}}{185}$ $+\frac{A_{Th}}{259}$ $+\frac{A_{K}}{4810}$
Iγ	Due to the various combinations of distinct natural activities in the sample, another index was proposed by a group of specialists to determine the amount of radiation hazard linked with the natural radionuclides in the samples.	$I_{\gamma }=\frac{A_{Ra}}{150}$ $+\frac{A_{Th}}{100}$ $+\frac{A_{K}}{1500}$
AGDE	The radioactive measure known as the yearly gonadal dose equivalent is used to calculate the doses of gamma radiation that are absorbed by the gonads.	AGDE (mSv y^−1^) = 3.09*A*_Ra_ + 4.18*A*_Th_ + 0.314*A*_K_
ELCR	The radioactive factor used to determine whether gamma radiation exposure caused lethal cancer is called excess lifetime cancer, where, Dl = Lifetime (70 years) and RF = cancer risk factor (0.05 Sv^−1^).	ELCR = AED_out_ × DL × RF

**Table 2:** Descriptive statistics of studied pegmatites.

	N	Mean	SD	Min	Max	Skewness	Kurtosis	CV, %
U-238 (Bq kg^−1^)	67	30.8	18.4	4.9	74.1	0.88	−0.16	60%
Th-232 (Bq kg^−1^)	67	27.3	26.0	0.8	129.9	1.54	2.79	95%
K-40 (Bq kg^−1^)	67	1045.5	366.9	219.1	1690.2	−0.41	−0.77	35%

**Table 3:** Comparison of ^238^U, ^232^Th and ^40^K activity concentration in the Hafafit Area area with numerous world studies.

Country	^238^U	^232^Th	^40^K	Reference
Egypt	30.8	27.3	1045.5	Present study
Egypt	137	82	1082	[19]
Saudi Arabia	28.82	34.83	665.08	[20]
Palestine	71	82	780	[21]
Jordan	41.52	58.42	897	[22]
India	25.88	42.82	560.6	[23]
Iran	77.4	44.5	1017.2	[24]
Spain	84	42	1138	[25]
Greek	74	85	881	[26]
Turkey	80	101	974	[27]
Nigeria	63.29	226.67	832.59	[5]

**Table 4:** Radium equivalent activity (Raeq), external hazard index (Hex), internal hazard index (Hin), absorbed dose rate (Dair), annual outdoor effective dose (AEDout), annual indoor effective dose (AEDin), and excess lifetime cancer (ELCR) in the pegmatite samples of the studied area.

Samples	Ra_eq_	H_in_	H_ex_	D_air_	AED_out_	AED_in_	AGDE	ELCR × 10^−3^
	(Bq/kg)			(nG/h)	(mSv)	(mSv)	(mSv)	
S1	97	0.3	0.3	47.3	0.06	0.2	0.34	0.20
S2	126	0.4	0.3	60.2	0.07	0.3	0.43	0.26
S3	138	0.5	0.4	62.5	0.08	0.3	0.43	0.27
S4	154	0.6	0.4	71.0	0.09	0.3	0.50	0.30
S5	171	0.6	0.5	79.1	0.10	0.4	0.56	0.34
S6	206	0.8	0.6	95.5	0.12	0.5	0.67	0.41
S7	272	0.9	0.7	125.4	0.15	0.6	0.89	0.54
S8	123	0.4	0.3	57.2	0.07	0.3	0.40	0.25
S9	160	0.6	0.4	71.8	0.09	0.4	0.50	0.31
S10	202	0.7	0.5	91.3	0.11	0.4	0.64	0.39
S11	208	0.7	0.6	94.4	0.12	0.5	0.66	0.41
S12	219	0.8	0.6	100.3	0.12	0.5	0.70	0.43
S13	140	0.5	0.4	64.5	0.08	0.3	0.46	0.28
S14	93	0.3	0.2	47.2	0.06	0.2	0.35	0.20
S15	94	0.3	0.3	47.8	0.06	0.2	0.36	0.21
S16	195	0.6	0.5	97.2	0.12	0.5	0.72	0.42
S17	182	0.6	0.5	87.8	0.11	0.4	0.63	0.38
S18	168	0.6	0.5	80.5	0.10	0.4	0.58	0.35
S19	152	0.5	0.4	77.9	0.10	0.4	0.58	0.33
S20	146	0.5	0.4	74.6	0.09	0.4	0.56	0.32
S21	115	0.4	0.3	59.2	0.07	0.3	0.44	0.25
S22	126	0.4	0.3	64.5	0.08	0.3	0.48	0.28
S23	151	0.5	0.4	77.2	0.09	0.4	0.58	0.33
S24	137	0.4	0.4	70.8	0.09	0.3	0.53	0.30
S25	129	0.4	0.3	65.8	0.08	0.3	0.49	0.28
S26	137	0.4	0.4	70.8	0.09	0.3	0.53	0.30
S27	120	0.4	0.3	62.0	0.08	0.3	0.47	0.27
S28	88	0.3	0.2	44.2	0.05	0.2	0.33	0.19
S29	139	0.4	0.4	71.1	0.09	0.3	0.53	0.31
S30	112	0.3	0.3	56.9	0.07	0.3	0.42	0.24
S31	92	0.3	0.2	45.5	0.06	0.2	0.33	0.20
S32	112	0.4	0.3	55.1	0.07	0.3	0.40	0.24
S33	122	0.5	0.3	59.1	0.07	0.3	0.42	0.25
S34	135	0.4	0.4	69.7	0.09	0.3	0.52	0.30
S35	104	0.4	0.3	50.5	0.06	0.2	0.37	0.22
S36	141	0.4	0.4	71.4	0.09	0.4	0.53	0.31
S37	99	0.3	0.3	48.8	0.06	0.2	0.36	0.21
S38	116	0.4	0.3	58.1	0.07	0.3	0.43	0.25
S39	132	0.4	0.4	65.6	0.08	0.3	0.48	0.28
S40	122	0.4	0.3	60.7	0.07	0.3	0.45	0.26
S41	112	0.4	0.3	56.1	0.07	0.3	0.41	0.24
S42	124	0.4	0.3	63.9	0.08	0.3	0.48	0.27
S43	124	0.4	0.3	65.2	0.08	0.3	0.49	0.28
S44	135	0.4	0.4	69.9	0.09	0.3	0.52	0.30
S45	127	0.4	0.3	65.3	0.08	0.3	0.49	0.28
S46	126	0.4	0.3	65.1	0.08	0.3	0.49	0.28
S47	131	0.4	0.4	67.2	0.08	0.3	0.50	0.29
S48	105	0.3	0.3	55.6	0.07	0.3	0.42	0.24
S49	96	0.3	0.3	49.8	0.06	0.2	0.37	0.21
S50	94	0.3	0.3	48.7	0.06	0.2	0.37	0.21
S51	94	0.3	0.3	48.5	0.06	0.2	0.36	0.21
S52	89	0.3	0.2	44.5	0.05	0.2	0.33	0.19
S53	192	0.6	0.5	93.3	0.11	0.5	0.68	0.40
S54	334	1.0	0.9	156.3	0.19	0.8	1.13	0.67
S55	216	0.6	0.6	105.8	0.13	0.5	0.78	0.45
S56	273	0.8	0.7	129.4	0.16	0.6	0.94	0.56
S57	182	0.5	0.5	87.4	0.11	0.4	0.64	0.38
S58	227	0.7	0.6	108.9	0.13	0.5	0.79	0.47
S59	135	0.5	0.4	65.2	0.08	0.3	0.47	0.28
S60	107	0.3	0.3	54.1	0.07	0.3	0.40	0.23
S61	176	0.6	0.5	85.4	0.10	0.4	0.62	0.37
S62	186	0.6	0.5	91.3	0.11	0.4	0.66	0.39
S63	193	0.7	0.5	94.6	0.12	0.5	0.69	0.41
S64	188	0.6	0.5	91.4	0.11	0.4	0.67	0.39
S65	211	0.7	0.6	102.2	0.13	0.5	0.74	0.44
S66	215	0.7	0.6	104.4	0.13	0.5	0.76	0.45
S67	209	0.7	0.6	100.6	0.12	0.5	0.73	0.43
Average	150	0.5	0.4	74	0.09	0.36	0.54	0.32
SD	50	0.2	0.1	22.5	0.03	0.11	0.16	0.10
Max	334	1.0	0.9	156.3	0.19	0.77	1.13	0.67
Min	88	0.3	0.2	44.2	0.05	0.22	0.33	0.19
GM	143	0.5	0.4	70.6	0.09	0.35	0.52	0.30

**Table 5:** Pearson correlation between natural radionuclides and the radiological hazards coefficients of pegmatite rocks, studied area, Egypt.

	U-238	Th-232	K-40	Ra_eq_	H_in_	H_ex_	D_air_	AED_out_	AED_in_	AGDE	ELCR
U-238	1										
Th-232	0.62	1									
K-40	−0.49	−0.17	1								
Ra_eq_	0.55	0.88	0.26	1							
H_in_	0.74	0.89	0.07	0.97	1						
H_ex_	0.55	0.88	0.26	1.00	0.97	1					
D_air_	0.49	0.82	0.37	0.99	0.94	0.99	1				
AED_out_	0.49	0.82	0.37	0.99	0.94	0.99	1.00	1			
AED_in_	0.49	0.82	0.37	0.99	0.94	0.99	1.00	1.00	1		
AGDE	0.43	0.78	0.44	0.98	0.92	0.98	1.00	1.00	1.00	1	
ELCR	0.49	0.82	0.37	0.99	0.94	0.99	1.00	1.00	1.00	1.00	1

## Data Availability

Not applicable.

## Supplementary Materials

The following supporting information can be downloaded at: https://www.mdpi.com/article/10.3390/toxics10100596/s1, Table S1. Values of eU (ppm), eTh (ppm), and K (%), as well as ^238^U, ^232^Th and ^40^K activity concentrations in the pegmatite for Hafafit area.
