# Supplementary material for: Natural Radioactivity Assessment and Radiation Hazards of Pegmatite as a Building Material, Hafafit Area, Southeastern Desert, Egypt
Source: Toxics. 2022 Oct 9;10(10):596. doi: 10.3390/toxics10100596 (PMC9607233; doi:10.3390/toxics10100596)
Supplement: Supplementary file 1 [file toxics-10-00596-s001.zip › toxics-1870937-supplementary.pdf]

Table S1. Values of eU (ppm), eTh (ppm), and K (%), as well as their values in (Bq.kg-1) for Hafafit pegmatite rocks.

Samples	eU (ppm)	eTh (ppm)	eK (%)
S1	2.3	4	1.9
S2	2.6	7	2.2
S3	4	12	0.8
S4	5	10	1.4
S5	4	13	1.9
S6	6	14	2.1
S7	**6**	22	2.9
S8	3	9	1.4
S9	5	14	**0.7**
S10	6	17	1.2
S11	5	19	1.5
S12	6	17	1.9
S13	3	12	1.4
S14	0.9	2	2.9
S15	1.1	2.3	2.8
S16	3.2	6	5
S17	4.1	8.5	3.4
S18	3.5	9.5	2.9
S19	2	2	4.8
S20	1.7	2.4	4.6
S21	1.3	1.2	3.8
S22	1.7	1.4	4
S23	1.8	2.2	4.8
S24	1.2	1.5	4.7
S25	2.5	1.1	3.8
S26	0.9	2.2	4.7
S27	1	2	4
S28	1.8	1.8	2.3
S29	1.4	2.7	4.4
S30	1.3	2.4	3.4
S31	2	2.4	2.2
S32	3	2.9	2.4
S33	4	3.8	2.1
S34	1	2	4.6
S35	2.1	4.7	2.1
S36	2	3	4.1
S37	1	5	2.4
S38	1.7	3	3.2
S39	2.1	4.1	3.4
S40	2.2	3.4	3.1
S41	2.1	2.8	2.9
S42	0.9	2	4.2
S43	1	**0.2**	4.6
S44	1.6	1.2	4.5
S45	1.4	1.9	4.1
S46	1.2	1.8	4.2
S47	1.2	2.8	2.4
S48	1.6	11.8	4.3
S49	1.5	32	5.4
S50	1.7	12	5.2
S51	2.2	22	4.9
S52	1.4	13	3.7
S53	3.4	14	4.3
S54	3	6	2.6
S55	1.3	2.8	3.1

S56	3	9	3.6
S57	4	7	4
S58	3.9	7.5	4.2
S59	3.6	8.5	3.9
S60	4.6	9.5	4.1
S61	4.1	10	4.4
S62	3.7	12	3.9
Average	2.6	7.2	3.3
SD	1.5	6.4	1.2
Max	6.0	32.0	5.4
Min	0.9	0.2	0.7
